# Impact of Mayo Adhesive Probability score and BMI on renal functional decline after robotic assisted partial nephrectomy

**DOI:** 10.1002/bco2.417

**Published:** 2024-08-30

**Authors:** Cesare Saitta, Marco Paciotti, Giovanni Lughezzani, Giuseppe Garofano, Margaret F. Meagher, Kit L. Yuen, Vittorio Fasulo, Roberto Contieri, Pier Paolo Avolio, Andrea Piccolini, Paola Arena, Matilde Mantovani, Edoardo Beatrici, Marta Calatroni, Francesco Reggiani, Rodolfo F. Hurle, Massimo Lazzeri, Alberto Saita, Paolo Casale, Ithaar H. Derweesh, Nicolò M. Buffi

**Affiliations:** ^1^ Department of Urology IRCCS Humanitas Research Hospital Rozzano Italy; ^2^ Biomedical Science Humanitas University Pieve Emanuele Italy; ^3^ Department of Urology UC San Diego Health System La Jolla California USA; ^4^ Nephrology and Dialysis Division IRCCS Humanitas Research Hospital Rozzano Italy

**Keywords:** BMI, CKD‐S, estimated glomerular filtration rate, functional decline, MAP score, obesity, renal cell carcinoma, survival analysis

## Abstract

**Purpose:**

The purpose of this study is to investigate the impact of Mayo Adhesive Probability (MAP) score and body mass index (BMI) on renal function decline after robotic assisted partial nephrectomy (RAPN).

**Methods:**

We queried our prospective database for patients who underwent RAPN between January 2018 and December 2023. Outcomes were development of de novo CKD‐S3 (estimated glomerular filtration rate [eGFR] < 60 ml/min/1.73 m^2^). Multivariable analysis (MVA) via Cox regression identified predictors for CKD‐S3. Kaplan–Meier Analyses was fitted for survival assessment. Finally, multivariable linear regression was utilized to identify predictors of delta eGFR at last follow‐up (preoperative eGFR—last eGFR).

**Results:**

Two‐hundred fifty‐eight patients were analysed (obese *n* = 49 [19%]; MAP score 0–2 = 135 [52.33%]; MAP score 3–5 = 123 [47.6%]) with a median follow‐up of 33 (IQR 20–42) months. MVA revealed, high MAP score (HR 2.29, *p* = 0.019), increasing RENAL score (HR 1.26, *p* = 0.009), increasing age (HR 1.04, *p* = 0.003), obesity (HR 2.38, *p* = 0.006) and diabetes mellitus (HR 2.38, *p* = 0.005) as associated with increased risk of development of CKD‐S3, while trifecta achievement was not (*p* = 0.63). Comparing low MAP score versus high MAP score 4‐year CKD‐S3 free survival was 87.8% versus 56.1% (*p* < 0.001). Multivariable linear regression showed that high MAP score (coefficient 6.64, *p* = 0.001) and BMI (coefficient 0.51, *p* = 0.011) were significantly associated with increased delta eGFR at last follow up.

**Conclusions:**

MAP score and increasing BMI are predictor for long term renal functional detrimental. These insights may call consideration for closer follow‐up or greater medical scrutiny prior surgery in obese patients and with elevated MAP score. Further investigations are requisite.

## 1 INTRODUCTION

Renal cell carcinoma (RCC) constitutes 4% of tumours in the United States, accounting for 14 890 deaths in 2023.^1^ The recent adoption of advanced imaging techniques and protocols has led to a downward stage migration for the diagnosis of RCC.^2^ While partial nephrectomy (PN) is the standard of care for cT1 renal masses,^3^, ^4^ the widespread use of the robotic platform has pushed the indications for PN towards more challenging settings, despite an increased risk of complication when compared to radical nephrectomy (RN).^5^, ^6^, ^7^, ^8^, ^9^, ^10^ Although PN has been associated with a better functional preservation and lower decline in estimated glomerular filtration rate (eGFR), non‐negligible proportions of patients nonetheless experience eGFR decline after PN.^9^, ^11^, ^12^, ^13^, ^14^, ^15^ On the other hand, the introduction of surgically induced chronic kidney disease (CKD‐S) has determined a paradigm shift in the decision making for the type of surgery (PN vs. RN), and an eGFR < 45 ml/min/1.73/m^2^ (CKD‐S3b) has been associated to worsened survival outcomes.^16^, ^17^ Moreover, up to 18.1% of patients with CKD‐3 (eGFR < 60 ml/min/1.73/m^2^) experienced rapid disease progression over 2 years.^18^ Thereby, identifying new predictors of renal functional decline is of outmost importance.^9^, ^19^, ^20^ While factors such as warm ischemia time (WIT), reduction in the remaining renal volume and ischemic injury to the preserved tissue during surgery are recognized as significant risk factors for post‐PN renal function, various metabolic elements have been associated with renal functional decline.^13^, ^16^, ^19^ In this nuanced scenario, the prominence of obesity (BMI > 30 kg/m^2^) and adherent perinephric fat (APF) have gained momentum. Mayo Adhesive Probability (MAP) score,^21^ an imaging‐based scoring system, was developed to anticipate the presence of APF before robotic‐assisted PN (RAPN). While MAP score has been used to assess durations and complexity of surgery, current literature lacks prospective evaluation of functional outcomes.^22^ Herein, we sought to elucidate the impact of MAP score and obesity on renal functional decline after RAPN with a particular focus on the progression towards moderate and severe chronic kidney disease (CKD).

## 2 PATIENTS AND METHODS

### 2.1 Study population

This is a single centre prospective study of patients who have undergone RAPN for a renal mass suspicious for RCC. Approval from the Institutional Review Board was obtained. All surgeries were exclusively executed by three experienced urologic oncology surgeons using a transperitoneal approach between January 2018 and December 2023. Arterial clamping was performed, and sliding clip technique was executed for renorrhaphy. Inclusion criteria included patients older than 18 years old affected by a localized renal mass eligible for RAPN. Patients with bilateral renal tumours, solitary kidney, synchronous metastases, end‐stage renal disease and those who received neoadjuvant treatment were excluded from the analysis. All patients underwent preoperative three‐dimensional (3D) abdominal CT scans or abdominal MRI to define the clinical stage and the anatomical characteristics of the tumour. All the radiological images were prospectively evaluated by an experienced urological surgeon (NMB) with the aim of assigning each variable included in the, RENAL,^23^ PADUA,^24^ SPARE^25^ and MAP^21^ scoring system. The MAP score was calculated according to Davidiuk, using two variables: posterior perinephric fat thickness and the presence of stranding. Posterior perinephric fat thickness was scored as follows: less than 1.0 cm = 0 points, 1.0–1.9 cm = 1 point and 2.0 cm or greater = 2 points. The presence of stranding was scored as follows: none = 0 points, Type 1 = 2 points and Type 2 = 3 points.^21^ Post‐operative follow‐up was conducted in accordance with the most contemporary clinical management protocols.^3^, ^4^, ^26^

### 2.2 Data collected

Demographic data included age at surgery, sex, American Society of Anesthesiologists Score (ASA), body mass index (BMI, kg/m^2^), presence of hypertension (HTN), diabetes mellitus (DM), tumour size, pathological stage,^27^ RENAL,^23^ PADUA,^24^ SPARE^25^ and MAP^21^ scoring system. Operative room variables analysed included estimated blood loss (EBL) and operative room time (OR). Surgical outcomes analysed were Margin Ischemia Complication (MIC)^28^ index and trifecta achievement. MIC was defined by the concurrent achievement of negative surgical margins, ischemia time <20 min and the absence of major complications (Clavien‐Dindo^29^ grade ≥ 3). Trifecta was defined by the simultaneous absence of positive surgical margins, major complications and a postoperative reduction in estimated glomerular filtration rate (eGFR) of less than 30%.^30^, ^31^ Serum creatinine was collected pre‐operatively and postoperatively at 3 months, 6 months and then annually thereafter. The CKD‐EPI equation was used to calculate eGFR.^32^ Delta eGFR was calculated through the following formula (preoperative eGFR—last eGFR). Postoperative acute kidney injury (AKI) was defined according to the Risk/Injury/Failure/Loss/Endstage (RIFLE) criteria (>25% reduction eGFR from baseline, to discharge).^33^ Follow‐up duration was calculated as the interval from surgery to the last recorded creatinine assessment. The primary outcome used was development of de novo surgically induced CKD‐S3 (eGFR < 60 ml/min/1.73 m^2^). As secondary outcomes, we endeavoured to align the predictive capabilities of the MAP score for the prediction of CKD‐S3 with existing nephrometric scores, aiming to integrate the MAP score into the current nephrometric scoring framework.

### 2.3 Statistical analysis

Patients were sub‐stratified by MAP score: low MAP score (0–2) and high MAP score (3–5). Descriptive analysis was carried out reporting median and interquartile range (IQR), and percentage for quantitative and qualitative variables, respectively. Continuous variables between groups were compared through the Wilcoxon rank sum test for non‐normal data, while categorical variables were analysed using the Pearson chi‐square test or the exact test of Fisher. Multivariable analysis (MVA), via Cox regression models, was tailored to elucidate predictors of outcome. Kaplan–Meier analysis (KMA) was employed for an evaluative comparison of the survival distributions. Differences between the survival distributions were evaluated with log‐rank test. A multivariable linear regression model was fitted to evaluate predictors of delta eGFR at last follow‐up. Finally, we combined the MAP score and the nephrometry score, which demonstrated the best predictive performance with respect to CKD‐S3 based on the Harrell's C‐index and the Akaike information criterion (AIC). Data were analysed using the Stata18.5/SE software package (StataCorp, Houston, United States), and alpha level was set at 0.05.

## 3 RESULTS

Overall, 258 patients were analysed (obese *n* = 49 [19%]; low MAP score *n* = 135 [52.3%]; high MAP score *n* = 123 [47.7%]) with a median follow‐up of 33 (IQR 20–42) months. Table 1 demonstrates demographics and clinical characteristics. Patients with high MAP score versus low MAP score had higher proportions of male (99 [80.5%] vs. 67 [49.6%], *p* < 0.001), DM (22 [17.9%] vs. 9 [6.7%], *p* = 0.006), HTN (73 [59.3%] vs. 58 [43%], *p* = 0.009) and post‐surgical AKI (29 [23.6%] vs. 18 [13.3%]). Furthermore, patients with high MAP score versus low MAP score had higher BMI (26.9 kg/m^2^ [24.5–29.8] vs. 25.2 kg/m^2^ [22.6–27.6], *p* < 0.001), higher OR time (146 min [117.0–185.0] vs. 130 min [110–158], *p* = 0.002) and EBL (100 ml [50–150] vs. 50 ml [30–100], *p* = 0.008). On the other hand, patients with low MAP score had higher preoperative eGFR (99.6 ml/min/1.73 m^2^ [91.8–106.9] vs. 94.6 ml/min/1.73 m^2^ [81.9–103.2], <0.001) and higher eGFR at last follow up (87.7 ml/min/1.73 m^2^ [75.7–98.8] vs. 71.1 ml/min/1.73 m^2^ [53.0–90.6], <0.001).

**TABLE 1:** Demographics and general clinical characteristics of the cohort

	Total	MAP score 0–2	MAP score 3–5	*p* value	Test
	*N* = 258	*N* = 135	*N* = 123		
Sex *n* (%)				<0.001	^a^
Female	92 (35.7%)	68 (50.4%)	24 (19.5%)		
Male	166 (64.3%)	67 (49.6%)	99 (80.5%)		
Age median year (IQR)	60.0 (52.0–68.0)	57.0 (50.0–64.0)	64.0 (57.0–71.0)	<0.001	^b^
BMI median kg/m^2^ (IQR)	25.9 (23.4–29.0)	25.2 (22.6–27.6)	26.9 (24.5–29.8)	<0.001	^b^
Hypertension *n* (%)				0.009	^a^
No	127 (49.2%)	77 (57.0%)	50 (40.7%)		
Yes	131 (50.8%)	58 (43.0%)	73 (59.3%)		
Diabetes *n* (%)				0.006	^a^
No	227 (88.0%)	126 (93.3%)	101 (82.1%)		
Yes	31 (12.0%)	9 (6.7%)	22 (17.9%)		
ASA score *n* (%)				0.12	^a^
1	191 (74.0%)	97 (71.9%)	94 (76.4%)		
2	32 (12.4%)	22 (16.3%)	10 (8.1%)		
3	35 (13.6%)	16 (11.9%)	19 (15.4%)		
Side *n* (%)				0.14	^a^
Right	130 (%)	74 (%)	56 (%)		
Left	128 (%)	61 (%)	67 (%)		
Clinical tumour size median mm (IQR)	30.0 (23.0–43.0)	30.0 (21.0–40.0)	32.0 (23.0–46.0)	0.089	^b^
RENAL score median (IQR)	7.0 (6.0–8.0)	7.0 (6.0–8.0)	7.0 (6.0–8.0)	0.27	^b^
RENAL score ≥ 7 *n* (%)				0.76	^a^
No	94 (36.4%)	48 (35.6%)	46 (37.4%)		
Yes	164 (63.6%)	87 (64.4%)	77 (62.6%)		
SPARE score median (IQR)	2.0 (1.0–4.0)	2.0 (0.0–3.0)	2.0 (1.0–5.0)	0.088	^b^
PADUA score median (IQR)	8.0 (7.0–9.0)	8.0 (7.0–9.0)	8.0 (7.0–9.0)	0.13	^b^
Pre‐Op Creatinine median mg/dl (IQR)	0.8 (0.7–0.9)	0.8 (0.7–0.9)	0.8 (0.7–1.0)	<0.001	^b^
Pre‐Op eGFR median^c^ (IQR)	97.5 (89.1–104.8)	99.6 (91.8–106.9)	94.6 (81.9–103.2)	<0.001	^b^
Pre‐Op CKD *n* (%)				0.011	^a^
No	238 (92.2%)	130 (96.3%)	108 (87.8%)		
Yes	20 (7.8%)	5 (3.7%)	15 (12.2%)		
Post‐Op eGFR median^c^ (IQR)	85.1 (69.5–99.6)	90.6 (77.0–102.0)	78.6 (62.4–95.9)	<0.001	^b^
Delta eGFR median^c^ (IQR)	13.5 (5.9–20.3)	12 (3.8–17‐7)	15.9 (6.4–31.4)	<0.001	^b^
AKI *n* (%)				0.033	^a^
No	211 (81.8%)	117 (86.7%)	94 (76.4%)		
Yes	47 (18.2%)	18 (13.3%)	29 (23.6%)		
Last eGFR median^c^ (IQR)	81.6 (64.1–94.6)	87.7 (75.7–98.8)	71.1 (53.0–90.6)	<0.001	^b^
Yes	54 (20.9%)	12 (8.9%)	42 (34.1%)		
Follow‐up median months (IQR)	33.0 (20.0–42.0)	34.0 (17.0–44.0)	31.0 (21.0–39.0)	0.42	^b^
OR time median minutes (IQR)	135.5 (113.0–168.0)	130.0 (110.0–158.0)	146.0 (117.0–185.0)	0.002	^b^
WIT median minutes (IQR)	12.0 (10.0–16.0)	12.0 (10.0–16.0)	14.0 (9.0–17.0)	0.39	^b^
EBL median ml (IQR)	70.0 (40.0–100.0)	50.0 (30.0–100.0)	100.0 (50.0–150.0)	0.008	^b^
Pathological tumour size median mm (IQR)	32.0 (23.0–40.0)	30.0 (21.0–38.0)	32.0 (24.0–42.0)	0.13	^b^
Pathological stage				0.18	^a^
Benign histology	64 (24.8%)	32 (23.7%)	32 (26.0%)		
1a	145 (56.2%)	79 (58.5%)	66 (53.7%)		
1b	19 (7.4%)	5 (3.7%)	14 (11.4%)		
2a	22 (8.5%)	14 (10.4%)	8 (6.5%)		
2b	1 (0.4%)	1 (0.7%)	0 (0.0%)		
3a	7 (2.7%)	4 (3.0%)	3 (2.4%)		
Histology *n* (%)				0.10	^a^
Adenoma	8 (3.1%)	6 (4.4%)	2 (1.6%)		
Angiomyolipoma	19 (7.4%)	11 (8.1%)	8 (6.5%)		
Cyst	4 (1.6%)	2 (1.5%)	2 (1.6%)		
Oncocytoma	33 (12.8%)	13 (9.6%)	20 (16.3%)		
ccRCC	144 (55.8%)	75 (55.6%)	69 (56.1%)		
chRCC	9 (3.5%)	2 (1.5%)	7 (5.7%)		
pRCC	26 (10.1%)	14 (10.4%)	12 (9.8%)		
vhRCC	15 (5.8%)	12 (8.9%)	3 (2.4%)		
Surgical margin *n* (%)				0.25	^a^
Negative	249 (96.5%)	132 (97.8%)	117 (95.1%)		
Positive	9 (3.5%)	3 (2.2%)	6 (4.9%)		
Trifecta achievement *n* (%)				0.068	^a^
No	45 (17.4%)	18 (13.3%)	27 (22.0%)		
Yes	213 (82.6%)	117 (86.7%)	96 (78.0%)		
MIC Achievement *n* (%)				0.73	^a^
No	46 (17.8%)	23 (17.0%)	23 (18.7%)		
Yes	212 (82.2%)	112 (83.0%)	100 (81.3%)		

Abbreviations: AKI, acute kidney injury; BMI, body mass index; ccRCC, clear cell renal carcinoma; chRCC, chromophobe renal cell carcinoma; CKD‐S3a, eGFR < 60^a^; CKD‐S3b, eGFR < 45^a^; EBL, estimated blood loss; eGFR, estimated glomerular filtration rate; MIC, margin ischemia complication; OR, operative room time; pRCC, papillary renal cell carcinoma; vhRCC, variant histology renal cell carcinoma; WIT, warm ischemia time.

^a^ Pearson's chi‐squared.

^b^ Wilcoxon rank sum.

^c^ ml/min/1.73/m^2^.

Table 2 illustrates MVA for predictors of de novo CKD‐S3. MVA revealed, high MAP score (HR 2.29, *p* = 0.019), increasing RENAL score (HR 1.26, p = 0.009), increasing age (HR 1.04, *p* = 0.003), obesity (HR 2.38, *p* = 0.006) and diabetes mellitus (HR 2.38, *p* = 0.005) as associated with increased risk of development of CKD‐S3 while trifecta achievement was not (*p* = 0.63).

**TABLE 2:** MVA for predictors of CKD‐S3

Predictor	Hazard ratio	95% CI	*P* > |*z*|
MAP (3–5 vs. 0–2)	2.29	1.15–4.57	0.019
RENAL score (continuous)	1.26	1.06–1.51	0.009
Age (continuous)	1.04	1.02–1.08	0.003
Obesity (yes vs. no)	2.38	1.28–4.43	0.006
Diabetes mellitus (yes vs. no)	2.38	1.29–4.39	0.005
Trifecta (yes vs. no)	0.85	0.45–1.62	0.637

Abbreviations: BMI, body mass index; CI, confidence interval.

**FIGURE 1:**
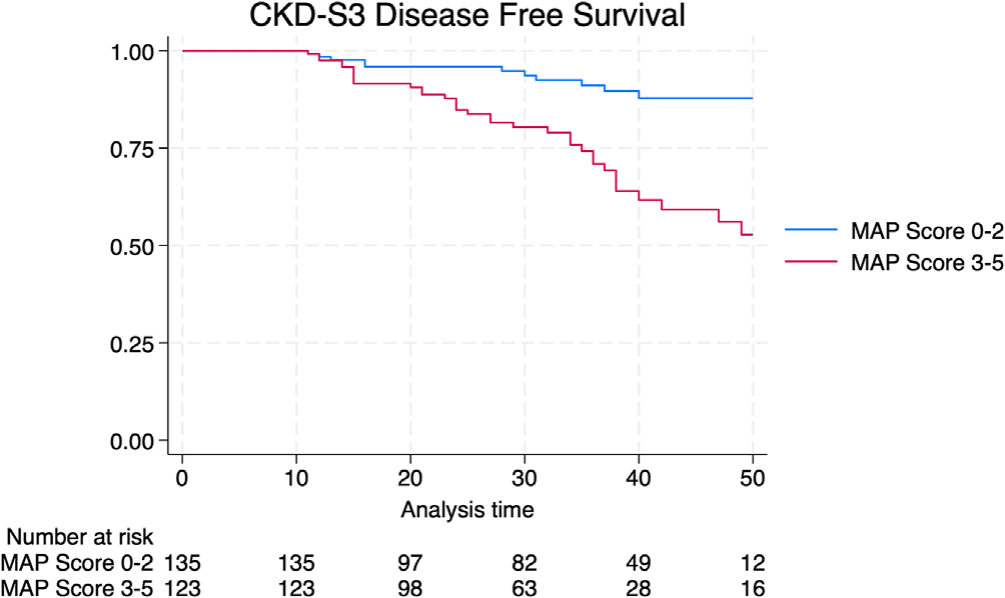
Kaplan–Meier curve for development of de novo CKD‐S3

Figure 1 illustrates Kaplan–Meier analyses. Comparing low MAP score versus high MAP score 4‐year CKD‐S3 free survival was 87.8% versus 56.1% (*p* < 0.001).

**FIGURE 2:**
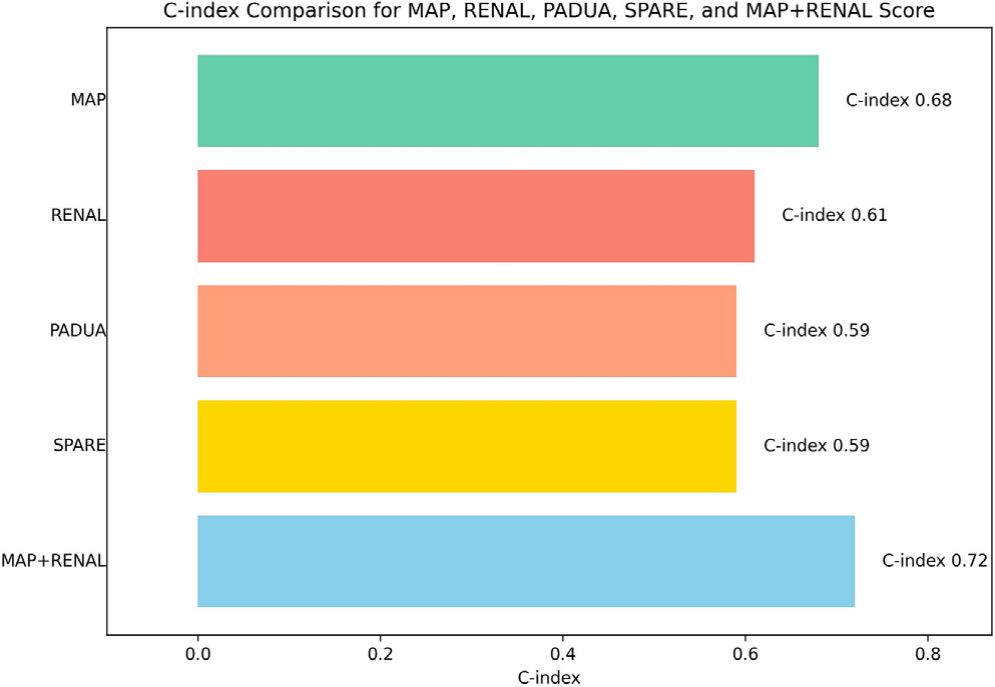
C‐Index analysis contrasting MAP, RENAL, SPARE and PADUA score for prediction of CKD‐S3

Multivariable linear regression showed that high MAP score (coefficient 6.64, *p* = 0.001) and BMI (coefficient 0.51, *p* = 0.011) were significantly associated with increased delta eGFR at last follow up. C‐index analysis for predictors of CKD‐S3 revealed MAP score as the best predictors among others nephrometric scores (C‐index: 0.68; Figure 2). The integration of high MAP score (3–5) and RENAL score ≥ 7 into a combined classification system has demonstrated enhanced predictive capabilities for CKD‐S3, surpassing the predictive performance of either the MAP or RENAL scores when used individually (MAP‐RENAL: C‐index 0.72, AIC 490.9; MAP: C‐index 0.68, AIC 494.65; RENAL: C‐index 0.61, AIC 508.382).

## 4 DISCUSSION

Adherent perinephric fat has been posited as risk factor for complex PN. Nonetheless, the association between MAP score and functional outcomes after RAPN has not been comprehensively elucidated. Using the largest prospective cohort so far present in the literature, we noted that high MAP score was associated with an increased risk of de novo CKD‐S3. On the other hand, our findings revealed that BMI and MAP score were linearly associated with worsened functional outcomes after RAPN. Furthermore, combining tumours and patients characteristics, we presented a new pragmatic and comprehensive tool that exhibited increased predictive performance to current scoring system for prediction of CKD‐S3. Our findings may guide informed decision making and foster strategies to protect renal function in this specific group of patients scheduled for RAPN.

Renal functional decline after RAPN is a quality‐of‐care concern, and impact of MAP score on long‐term renal functional decline has not well established in existing literature. Hata et al.^22^ conducted a retrospective study using a dataset of 78 laparoscopic PN. The authors defined a decrease in the eGFR preservation rate to 90% or less at 3 months post‐surgery as an indicator of renal function deterioration. In their cohort, 47.4% of patients experienced a deterioration in renal function after PN and upon univariate logistic regression MAP score was not associated with renal functional decline (*p* = 0.99), while interestingly, the authors found that MAP score on the unaffected side was associated with the outcome of interest (OR 1.38 *p* = 0.02). Compared to Hata et al.,^22^ our study derives strength from a larger sample size (258 vs. 78 patients) and the use of the Cox proportional hazard model instead of a binary logistic regression model. This decision hinges upon the salient nature of the ‘time‐to‐event’ variable in our dataset, which denotes the time until the onset of CKD, thereby augmenting the analytical rigour of our investigation. While the preceding study delineated renal functional decline as an eGFR preservation below 90% within 3 months post‐surgery, our study boasts an extended follow‐up duration of 33 months. Furthermore, we employed a more stringent outcome measure, defining significant renal function deterioration as an eGFR < 60 ml/min/1.73 m^2^. In this respect, our MVA revealed that MAP score was associated with increased risk of de novo CKD‐S3 (HR 2.29, *p* = 0.019) as well as our KMA, which revealed worsened CKD‐3 disease free survival in patients with high MAP score (87.8% vs. 56.1% [*p* < 0.001]). Cumulatively, these findings suggest that the presence of high MAP score and APF may amplify the inflammatory status, which could impair renal function via the paracrine effects of adipocytokines.^34^

Indeed, while obesity appears to paradoxically serve as a protective factor against adverse oncological outcomes in RCC, a controversial phenomenon known as the ‘obesity paradox’, its influence on renal functional decline remains undeniably significant.^35^ Rosen et al.^36^ performed a retrospective analysis of 1770 (obese *n* = 529 [45.2%]) RAPN, investigating the relationships between obesity and AKI. Main drivers for AKI upon discharge in their MLR were obesity (OR 1.81, *p* = 0.031), male sex (OR 1.54, *p* = 0.028) and increasing tumour size (OR 1.23, *p* < 0.001). Interestingly, the authors reported that a BMI exceeding the normal weight range did not show a relationship with a greater decline in eGFR per month after RPN. Similarly, Richards et al.^37^ evaluated the effect of BMI on renal functional decline using a retrospective database of 235 patients with a median follow‐up of 29 months (IQR 19–45). Their MLR model showed Class 1 obesity (BMI 30–35 kg/m^2^; OR 4.68, *p* = 0.019), Class 2 obesity or above (OR 4.27, *p* = 0.033) and age (OR 1.06, *p* = 0.014) correlated with a heightened risk of CKD ≥ 3 (eGFR < 60 ml/min/1.73/m^2^), while their multivariable linear regression model revealed that BMI was significantly linked to an absolute decrease in eGFR at 1 year (0.38 ml/min/1.73 m^2^ eGFR reduction for every 1 kg/m^2^ increase in BMI, *p* = 0.009). Consistent with the observations of Rosen et al.^36^ and Richards et al.,^37^ our study corroborated the association between obesity and heightened risk of CKD‐S3 (HR 2.38, *p* = 0.006) and revealed a linear relationship between increasing BMI and increased delta eGFR at the final follow‐up (coefficient 0.51, *p* = 0.011). Taken together, these insights underline the critical need for targeted strategies in managing obesity preoperatively, to mitigate its detrimental effects on renal function in RCC patients.

On the other hand, nephrometric scores serve as valuable preoperative tools tailored to the anatomical features of neoplasm. In contrast, MAP score focuses on patient‐specific anatomical characteristics, particularly in predicting the presence of APF. While previous studies have recognized the association between the MAP score and the RENAL score, there remains a limited characterization of how a combination of these scores can predict functional outcomes. To the best of our knowledge, Jin et al.^38^ were the first to combine RENAL and MAP score to evaluate the potential predictive value for intraoperative outcomes in retroperitoneal laparoscopic PN. In their cohort of 293 (intraoperative complication *n* = 21 [7.5%]), MAP and RENAL scores demonstrated robust predictability for intraoperative complications, with individual area under the curve (AUC) of 0.728 and 0.759, respectively. Upon amalgamating these two scores, the AUC for overall intraoperative complications exhibited a notable improvement (AUC = 0.847). In akin manner to our colleagues, we attempted to elucidate impact of RENAL and MAP score on CKD‐S3. Indeed, our findings revealed increasing prognostic capability when we amalgamate RENAL and MAP score (MAP‐RENAL: C‐index 0.72, AIC 490.9; MAP: C‐index 0.68, AIC 494.65; RENAL: C‐index 0.61, AIC 508.382). Taken together, these findings underscore the importance of jointly considering both RENAL and MAP score in predicting adverse renal perioperative complications and long‐term functional outcomes. These findings prompt consideration towards a more comprehensive preoperative scenario, which should consider both anatomical tumour and patients feature as well.

While our study provides valuable insights, it is not exempt from limitations. The study's single‐centre design, based in a tertiary referral centre, may restrict the generalizability of our findings to smaller healthcare settings or diverse patient populations. Despite the surgeries being performed by experienced urologic oncology surgeons, the potential for inter‐operator variability remains a concern. However, surgeons employed the same technique for each surgical procedure, thus reducing a potential source of bias. Additionally, the stringent surgical criteria for obese patients with comorbidities could introduce selection bias, excluding individuals with less resilience or challenging health conditions, impacting the external validity of our study. While we controlled for relevant clinical variables, the presence of unaccounted confounders might influence the observed outcomes. Furthermore, the relatively small number of patients who experienced CKD‐S3 (54, 20.9%) does not provide us with the robustness required to adjust for all possible confounders of the outcome. Therefore, while the findings offer valuable insights, they should be interpreted with caution, as the possibility of residual confounding cannot be entirely ruled out. The lack of a control group undergoing a different surgical procedure and the limited granularity of the database hinder our capacity for direct comparisons, potentially constraining the depth of our analysis, particularly in comparison to a RN cohort. Furthermore, the relatively short median follow‐up duration of 33 months may not fully capture long‐term outcomes. Another significant limitation is the potential inter‐observer variability in the assignment of the MAP score, as the score was assessed by a single urologist, as such does not allow quantitively statements to be made in this respect. However, the assessment of the MAP score by an experienced urologic surgeon enhances the consistency of our data, as the surgeon's expertise ensures more accurate and consistent evaluations. Nonetheless, the significant outcomes of expedited functional decline associated with high MAP score and obesity ought to encourage the contemplation of strategies to adopt a multidisciplinary approach to prevent renal injury in patients with those characteristics undergoing RAPN.

## 5 CONCLUSION

Presence of high MAP score and increasing BMI is independent risk factor associated with renal functional decline below threshold of CKD‐S3. Furthermore, by combining preoperative MAP and RENAL scores, we have developed a novel score that may identify a subgroup of patients at risk for adverse functional outcomes. These patients could benefit from closer follow‐up and increased medical scrutiny. Further investigations are requisite.

## AUTHOR CONTRIBUTIONS

Cesare Saitta, Marco Paciotti, Ithaar H. Derweesh, Nicolò M. Buffi, Rodolfo Hurle, Alberto Saita, Massimo Lazzeri and Giovanni Lughezzani conceived and designed the study. Giuseppe Garofano, Vittorio Fasulo, Roberto Contieri, Pier Paolo Avolio, Andrea Piccolini, Paola Arena, Matilde Mantovani, Francesco Reggiani, Marta Calatroni and Edoardo Beatrici performed the database search, study selection and data extraction. Cesare Saitta, Margaret F. Meagher and Kit L. Yuen conducted the statistical analysis. All authors interpreted data for the work. Cesare Saitta drafted the article. All authors reviewed and revised the article. All authors read and approved the final article.

## CONFLICT OF INTEREST

There are no potential competing interests to declare.
